# Environmental determinism, and not interspecific competition, drives morphological variability in Australasian warblers (Acanthizidae)

**DOI:** 10.1002/ece3.3925

**Published:** 2018-03-23

**Authors:** Vicente García‐Navas, Marta Rodríguez‐Rey, Petter Z. Marki, Les Christidis

**Affiliations:** ^1^ Department of Integrative Ecology Estación Biológica de Doñana (EBD‐CSIC) Seville Spain; ^2^ Department of Biosciences Swansea University Swansea Wales UK; ^3^ Center for Macroecology, Evolution and Climate Natural History Museum of Denmark University of Copenhagen Copenhagen Denmark; ^4^ Natural History Museum University of Oslo Oslo Norway; ^5^ National Marine Science Centre Southern Cross University Lismore NSW Australia; ^6^ School of BioSciences University of Melbourne Parkville Vic. Australia

**Keywords:** acanthizids, allopatric, climate, foraging niche, macroevolution, morphological convergence, passerines, phenotypic landscape

## Abstract

Interspecific competition is thought to play a key role in determining the coexistence of closely related species within adaptive radiations. Competition for ecological resources can lead to different outcomes from character displacement to, ultimately, competitive exclusion. Accordingly, divergent natural selection should disfavor those species that are the most similar to their competitor in resource use, thereby increasing morphological disparity. Here, we examined ecomorphological variability within an Australo‐Papuan bird radiation, the Acanthizidae, which include both allopatric and sympatric complexes. In addition, we investigated whether morphological similarities between species are related to environmental factors at fine scale (foraging niche) and/or large scale (climate). Contrary to that predicted by the competition hypothesis, we did not find a significant correlation between the morphological similarities found between species and their degree of range overlap. Comparative modeling based on both a priori and data‐driven identification of selective regimes suggested that foraging niche is a poor predictor of morphological variability in acanthizids. By contrast, our results indicate that climatic conditions were an important factor in the formation of morphological variation. We found a significant negative correlation between species scores for PC1 (positively associated to tarsus length and tail length) and both temperature and precipitation, whereas PC2 (positively associated to bill length and wing length) correlated positively with precipitation. In addition, we found that species inhabiting the same region are closer to each other in morphospace than to species outside that region regardless of genus to which they belong or its foraging strategy. Our results indicate that the conservative body form of acanthizids is one that can work under a wide variety of environments (an all‐purpose morphology), and the observed interspecific similarity is probably driven by the common response to environment.

## 1 INTRODUCTION

The way in which species interact can promote evolutionary divergence of ecomorphological traits, thereby acting as an engine to generate species differences in adaptive radiations (Schluter, 2000). This assertion relies on the idea that the strength of competition increases with increasing taxonomic (and, hence, phenotypic) similarity between competitors, an idea dating back to the *Origin of Species* where Darwin proposed his “principle of divergence of character” to explain how species arise and why they differ from one another morphologically (Darwin, 1859). According to Darwin's claim, when organisms compete for limited resources, competitively mediated selection should favor those individuals that are least like their competitors. Consequently, lineages undergoing this selective pressure should become more dissimilar over time. Lack (1947) was the first in suggesting a natural scenario to examine the role of competition as mechanism for promoting adaptive diversification using the Darwin's finches from the Galápagos archipelago as model system. In his seminal work, Lack introduced the method of comparing sympatric and allopatric populations for this purpose. A decade later, Brown and Wilson (1956) redefined the concept of divergence of character and coined the term “character displacement,” a process by which sympatric species evolve divergent morphologies in order to minimize competition by, for instance, specializing on different foraging niches. According to Brown and Wilson (1956), character displacement may arise as a consequence of natural selection favoring in each population those individuals whose phenotype allows them to exploit resources not used by members of other species (Grant, 1972; reviewed in Pfennig & Pfennig, 2010). Since then an overwhelming body of literature has focused on identifying the conditions that promote character displacement, some of them using elegant approaches such as the sister‐lineage method (also known as the Noor's method; Noor, 1997; Martin, Montgomerie, & Lougheed, 2010) or laboratory experiments (e.g., Bailey & Kassen, 2012). Although some well‐studied systems (*Anolis* lizards, three‐spined sticklebacks, Darwin's finches) have provided strong evidence in support of the character displacement idea (e.g., Davies, Meiri, Barraclough, & Gittleman, 2007; Grant & Grant, 2006; Grant & Grant 2008; Losos, 1990; Schluter & McPhail, 1992), most studies found no conclusive evidence for this phenomenon (reviewed in Stuart & Losos, 2013). Thus, it seems that a process exists preventing character displacement (morphological divergence) in most circumstances. Such process could be the effect that shared local conditions exert upon coexisting species, which favors morphological convergence. Yet, the relative importance of these two opposing selective pressures (i.e., climate‐provoked morphological resemblance *versus* competition‐driven morphological divergence) remains poorly explored (Bothwell, Montgomerie, Lougheed, & Martin, 2015).

Ecomorphological studies typically focus on the relationships between a species’ morphology and its environment at local spatial scales (e.g., Losos, Warheit, & Schoener, 1997). Although some of these studies have documented striking cases of morphological adaptation, most have reported an absence of well‐defined ecomorphological relationships (e.g., Maestri et al., 2017). An drawback of these studies is that the fine spatial scale limits inferences that can be made in relation to the broadscale patterns of variation (i.e., across the distribution range of species), which are important in order to fully understand factors underlying morphological diversification in organisms with high dispersal capacity. Thus, studies addressing morphological diversification at both small and large scales are timely and necessary, especially in a global environmental change scenario. This approach is now feasible by means of GIS data that allow us to characterize the environmental attributes of each species’ niche and thus, to test whether relationships between a species’ morphology and environmental factors can be detected across continental spatial scales (Kozak, Graham, & Wiens, 2008; Miller, Wagner, Harmon, & Ricklefs, 2017).

**Figure 1:**
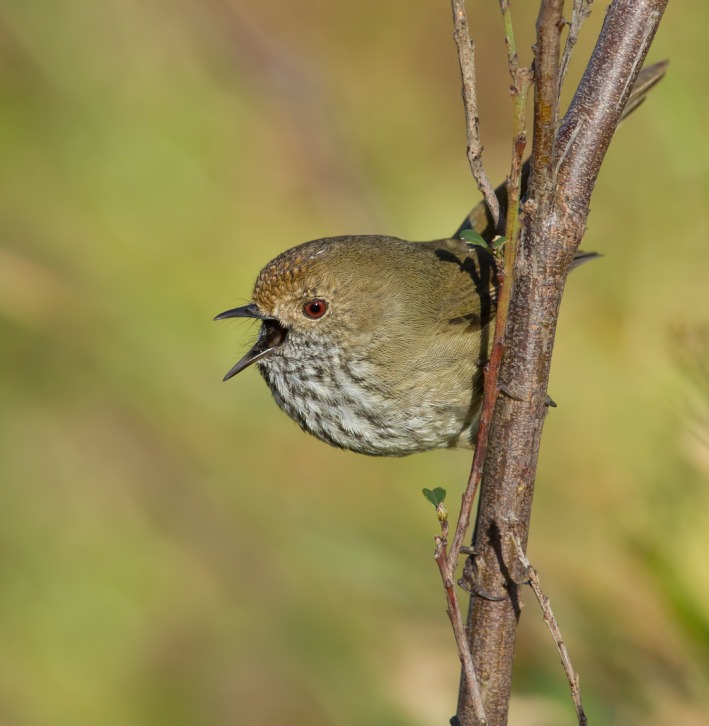
Brown thornbill (*Acanthiza pusilla*), one of the acanthizid species included in the study. Photograph: Richard Hall

In this study, we aimed to test the hypothesis that the coexistence of ecologically similar species promotes competitive interactions that lead to ecomorphological divergence using the songbird family Acanthizidae as a study case. The Acanthizidae is largely restricted to Australia and New Guinea. Only the genus *Gerygone* has spread into New Zealand and several South Pacific Islands, and west into the Lesser Sundas (two species) and Southeast Asia (one species). This family comprises 64 species of small warbler‐like passerines (Figure 1) that can be subdivided into three main groups corresponding to the three most species‐rich genera: *Gerygone* (19 species of gerygone), *Acanthiza* (14 species of thornbill), and *Sericornis* (12 species of scrubwren). This taxonomic group is ideal for the purpose of our study as it comprises both allopatric and sympatric lineages. *Gerygone* is a genus in which evolution has mainly taken the form of specialization to different habitats (from rainforests and mangroves to semiarid woodlands and sandy plains), to produce a largely allopatric assemblage of species (Keast & Recher, 1997; Nyari & Joseph, 2012). Most gerygones are canopy‐gleaners (they obtain their prey by gleaning and snatching it from the foliage), which implies that they exploit the same foraging niche. In *Acanthiza* and *Sericornis*, by contrast, several species co‐occur using different foraging heights or substrates. For instance, striated thornbills (*Acanthiza lineata*) glean from foliage in the canopy, while brown thornbills (*A. pusilla*) forage among shrubs and buff‐rumped thornbills (*A. reguloides*) often feed on ground foliage (Gregory, 2007). The yellow‐throated scrubwren (*Sericornis citreogularis*) coexists with the Atherton scrubwren (*S. keri*), large‐billed Scrubwren (*S. magnirostra*), and white‐browed scrubwren (*S. frontalis*) in the same remnant forests of the Australian east coast, whereas the large scrubwren (*S. nouhuysi*), Papuan scrubwren (*S. papuensis*), buff‐faced Scrubwren (*S. perspicillatus*), and bicolored mouse‐warbler (*Crateroscelis nigrorufa*) form a sympatric assemblage of species in New Guinea (Christidis, Schodde, & Baverstock, 1988; Diamond, 1972, 1973; Keast, 1978). Hence, a priori it should be expected to find greater morphological disparity in the two sympatric groups (thornbills and scrubwrens) as niche (morphological) partitioning would lessen competitive interactions and thereby, facilitate coexistence.

We first examined the existence of differences among the three main clades in terms of degree of morphological resemblance and extent of range overlap. Subsequently, we explored the adaptive landscape of acanthizids using the SURFACE algorithm (Ingram & Mahler, 2013), which uses a stepwise‐modeling approach to first identify peak shifts and then to identify whether any of these shifts involve convergence toward the same peaks (see e.g. Mahler et al. 2013; Davis et al. 2014; Astudillo‐Clavijo et al. 2015). The final model obtained from SURFACE was compared to alternative evolutionary models (Brownian Motion, single‐optimum Ornstein‐Uhlenbeck, and early‐burst) in order to determine those factors correlated with the phenotypic optima or evolutionary rates of the traits. We then compared the morphological distance to the phylogenetic distance of each species pair using Mantel tests. When distantly related taxa are morphologically similar, it is interpreted as strong evidence for evolutionary adaptation (Stayton, 2015). We also examined the correlation between morphological divergence and range overlap to test the influence of between species interactions on morphology. Lastly, we integrated climate and species distribution data to characterize species’ abiotic requirements at large scale, and thereby to examine the relationship between environmental (climatic) and morphological traits in a phylogenetic framework (i.e., how phenotypes vary across environments). In this way, we assessed whether similar environmental conditions lead to similar morphotypes, which would support the scenario of environment‐driven prevention of character displacement in morphology (Gvoždík, Moravec, & Kratochvíl, 2008; Martin & Meehan, 2005).

## 2 MATERIAL AND METHODS

### 2.1 Morphological, ecological, and phylogenetic data

We compiled morphological data from the literature, mainly from the *Handbook of Australian, New Zealand and Antarctic Birds* (Higgins & Peter, 2002) and the *Handbook of New Guinea Birds* (Rand & Gilliard, 1967) for a total of 53 taxa accounting for 82% of currently recognized Acanthizidae species (Dickinson & Christidis, 2014). Although our focus was on the Australian and New Guinean regions, where all three genera (*Acanthiza*,* Sericornis*,* Gerygone*) co‐occur, we did include the two New Zealand centered species of *Gerygone* to provide a contrast. In addition, we included the monotypic basal subfamily Pachycareinae (*Pachychare*) as outgroup.

The three genera are each part of major clades sometimes treated as subfamilies: Acanthizinae, Gerygoninae, and Sericornithinae (Schodde & Christidis, 2014). We included species from all the genera in these subfamilies to provide a broader perspective and increase the power of our analyses. Specifically, we gathered information (mean values for males of the nominal subspecies) for the following morphological traits: body size, wing length, tarsus length, tail size, and bill length. These traits are strongly associated with ecological and behavioral characteristics such as diet and substrate utilization. Wing morphology correlates with dispersal ability (Fitzpatrick, 1985; Kennedy et al., 2016); tarsus length is tightly associated with foraging mode and prey capture modes (Leisler 1980; Carrascal, Moreno, & Tellería, 1990; García‐Navas, Rodríguez‐Rey, & Christidis, 2018; Thomas, 1997); tail length has a strong influence on foraging movements due to its aerodynamic properties in terms of maneuverability and stability (Thomas & Balmford, 1995); and bill size has been shown to correlate with prey size and attacking behavior (Grant & Grant, 2006; Lederer, 1972). These variables were corrected for body size and from the obtained size‐corrected values (i.e., relative wing length, relative tarsus length, relative tail length, and relative bill length), we performed a principal component analyses (PCA) in order to obtain a set of uncorrelated variables. The PCA yielded two critical principal component (PC) axes that explained ~72% of the overall variation (PC1 = 45.3% and PC2 = 26.9%). The highest morphological loadings from PC1 were tarsus length and tail length (positively loaded: 0.89 and 0.81, respectively), whereas PC2 was most strongly influenced by wing length and bill length (factor loadings: 0.76 and 0.86, respectively). Species scores on PC axes were used as the input in subsequent comparative analyses. We obtained very similar results using a phylogenetic‐corrected approach (phylogenetic PCA, *p*PCA) as alternative method (Revell, 2009). However, as it has been recently suggested that use of pPCA may bias inference toward identifying particular evolutionary patterns and thus, may be misleading (Uyeda, Caetano, & Pennell, 2015; see also Bookstein, 2012), we only show the results based on the PCA analysis for the sake of brevity.

**Figure 2:**
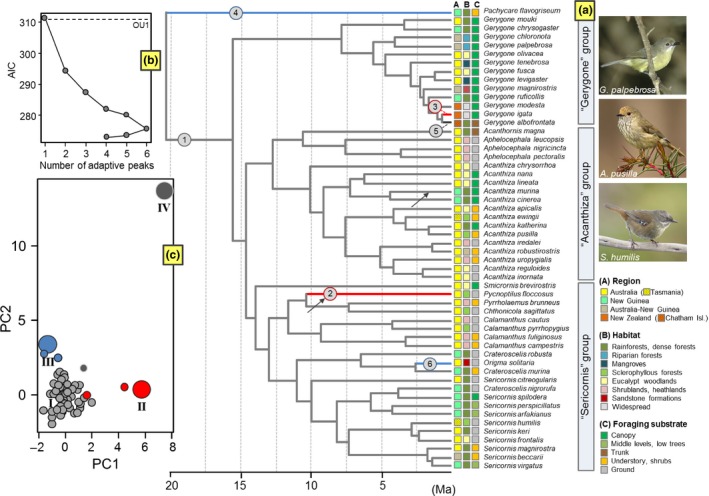
Results of SURFACE analyses. (a) Chronogram derived from Marki et al. (2017) with branches colored according to the selective regime estimated from the best‐fit model (convergent regimes are color‐mapped whereas nonconvergent regimes are in gray‐scale). Numbers on branches indicate the order in which regime shifts were added in the forward phase. Arrows denote the position of regime shifts identified when performing SURFACE analyses for each clade separately (see main text). The bottom‐inset (b) shows change in the corrected Akaike’ Information Criterion (AIC_c_) during the forward and backward phases of SURFACE analysis. The dashed line indicates the AIC_c_ for the single‐peak Ornstein‐Uhlenbeck (OU1) model. The AIC_c_ corresponding to the BM model is out of range (AIC_c_ = 323.1) and thus it is not shown. The top‐inset (c) illustrates the position of adaptive peaks (numbered using Roman numerals) in functional morphospace based on the best model (large circles: peaks; small circles: species scores). All pictures are Creative Commons

Data on distribution of acanthizid species were obtained from the IUCN Red List of Threatened Species (IUCN 2017). From these range maps, we characterized both the temperature and precipitation profiles (i.e., climatic preferences) for each species by subtracting mean annual temperature and annual precipitation for each occupied grid‐cell (at a resolution of 100 × 100 km) in QGIS v.2.18.12 (http://www.qgis.org) from climate layers (BIO1 and BIO12, respectively) in the WorldClim database (http://www.worldclim.org). After exploring interrelationships among the 19 bioclimatic variables available in WorldClim, we chose to use mean annual temperature and annual precipitation to characterize climate space for the sake of simplicity. Acanthizid species were then classified into different categories according to the region they inhabit (five categories), their main habitat (eight categories), and foraging niche (five categories) (see Figure 2). Information on habitat and niche preferences were obtained from Higgins and Peter (2002) and Gregory (2007).

Concerning phylogenetic data, we obtained an estimate of the phylogenetic relationships among the 53 acanthizid species included in this study, from Marki et al. (2017). In this recent publication, Marki et al. (2017) used a supermatrix approach including five mitochondrial (*12S*,* cyt‐b*,* COI*,* ND2,* and *ND3*) and four nuclear markers (*Fib‐5*,* GAPDH*,* RAG‐1,* and *RAG‐2*) to infer a time‐calibrated phylogeny of the infraorder Meliphagides radiation, which is divided into five families including the Acanthizidae. For more information about phylogenetic methods, we refer to the study by Marki et al. (2017).

### 2.2 SURFACE analyses

We used SURFACE to identify rate shifts and convergence of phenotypic optima on the phylogeny (Ingram & Mahler, 2013; Mahler & Ingram, 2014). This method detects cases of phenotypic convergence under an Ornstein‐Uhlenbeck (OU) process of evolution (also known as Hansen's model; Hansen, 1997; Butler & King, 2004). SURFACE analyses consist of two distinct phases: a “forward” phase starting with a single‐peak during which regime shifts are added to the phylogeny until there is no further improvement to the model, and a “backward” phase in which shifts toward the same peaks are identified and collapsed (this step is iterated until AIC_c_ scores cease to improve). An advantage of this method is that by taking as input only the phylogeny and multidimensional phenotypic data, it can identify cases of convergence across a clade while avoiding potential biases associated with the subjective a priori designations of candidate convergent taxa (Mahler & Ingram, 2014). At this point, it should be noted that convergence is not necessarily indicative of deterministic evolution (i.e., adaptation to specific ecological conditions) (Speed & Arbuckle, 2016; Stayton, 2008). Convergence may arise due to specific mechanisms such as adaptation in response to the same selective pressures (process‐based convergence), or as result of undirected evolution. Here, we refer to convergence as the evolution of different regimes toward the same adaptive peak with no necessary assumption of any particular process (Stayton, 2015). We ran SURFACE on PC1 and PC2 jointly on a sample of 100 posterior distribution trees. In order to determine to what extent convergence in the adaptive landscape of functional morphology in acanthizids could have occurred by chance under a nonconvergent process, we compared the fit of the convergent SURFACE model with a simpler initial, single‐peak Ornstein‐Uhlenbeck model (OU1). Furthermore, we compared the fit of the final SURFACE model to a Brownian motion (BM) model. In this way, we tested whether the same number of adaptive peaks could have also resulted from a random‐walk process (Arbour & López‐Fernández, 2014; Ingram & Mahler, 2013). We also ran SURFACE analyses separately for each one of the three main clades or subfamilies (Gerygoninae, Acanthizinae, and Sericorninae) in order to test if the number of evolutionary regimes and the location of regime shifts vary to a greater or lesser extent when performing these analyses at a smaller scale (that is, using a reduced dataset). In addition, we assessed the overall responsiveness of acanthizid lineages to the inferred selective regimes, which was quantified in terms of phylogenetic half‐life (*t*_1/2_). A phylogenetic half‐life that is long relative to the total depth of the phylogeny indicates slow evolution toward phenotypic optima and can contribute to morphological diversity among species that share a selective regime when they have evolved under that regime for different amounts of time (e.g., Collar, Schulte, & Losos, 2011).

### 2.3 Evolutionary model fitting

We compared the fit of the best SURFACE model to three evolutionary models that lack deterministic convergence and to two models with a priori designation of selective regimes based on foraging niche categories and geographic distribution (region). Specifically, to each PC axis, we fitted the following models using maximum‐likelihood inference: (1) a BM model in which traits evolve following a random‐walk process and morphological disparity accumulates roughly linearly through time (due to randomly fluctuating selection or genetic drift) (Felsenstein, 1985); (2) an early‐burst (EB) or adaptive radiation model in which phenotypic change occurs rapidly after lineages enter available niches and decreases as niches are filled (Harmon et al., 2010; Simpson, 1944); (3) a single‐peak OU model (OU1) with one parameter for the variance of random‐walk (σ^2^) and strength of selection (α) toward a global optimum for all acanthizids (Butler & King, 2004); (4) a multi‐peak OU model (OUM_region_) with separate random‐walk variances for each geographic region (Australia, New Guinea, Australia‐New Guinea, New Zealand, and Chatham Islands); (5) a multi‐peak OU model (OUM_niche_) with separate random‐walk variances for each one of the five foraging niche categories (“canopy,” “low trees,” “trunks,” “shrubs,” and “ground”) and one global selection parameter (α); and (6) a multi‐peak OU model (OUM_SURFACE_) with separate random‐walk variances for each one of the adaptive peaks identified using SURFACE (see Section 2.1). To deal with phylogenetic uncertainty, the BM, EB, and OU1 models were run across a sample of 100 trees obtained from the posterior distribution of the Bayesian analysis. For the multi‐peak (OUM) models, we first built stochastic character‐mapped reconstructions (SIMMAP; Bollback, 2006) of (1) foraging niche categories, (2) regions, and (3) adaptive peaks estimated by SURFACE, for each of the 100 trees sampled from the posterior distribution, using *phytools* (Revell, 2012). Models were implemented using the R packages *geiger* (Harmon, Weir, Brock, Glor, & Challenger, 2008) and *OUwie* (Beaulieu & O'Meara, 2014) and compared by means of the sample size‐corrected Akaike's Information Criterion (AIC_c_).

### 2.4 Mantel tests

We used Mantel tests to determine whether more similar species are those that (1) are closely related; (2) are more geographically close; (3) do not exhibit range overlap; and/or (4) share climatic conditions. To this end, we first produced matrices representing the phylogenetic, morphological, climatic, and geographic distances between all pairs of species. The phylogenetic matrix represents the patristic distance between each pair of species in the phylogeny depicted in Marki et al. (2017). Patristic distances were obtained using the function cophenetic in the *stats* package (R Core Team 2017). For the morphological matrix, we computed the Euclidean distance for all pairwise comparisons between species in the space defined by the two PC axes (PC1 and PC2). To compute the matrix of geographic distances, we first obtained the distributional midpoint of each species from a presence–absence matrix using the R package *letsR* (Vilela & Villalobos, 2015). In addition, as information based on single location is not useful to quantify overlap (sympatry) between each pair of species, we also computed a range overlap matrix from our presence–absence matrix using the function “lets.overlap” in the R package *letsR* (Vilela & Villalobos, 2015). Range overlap represents the proportion of the smaller range that occurs within the larger range (Cheeser & Zink, 1994; Martin et al., 2010) so that values range from 0 (no overlap) to 1 (smaller range completely overlapped by the larger range). The climatic matrix was constructed as the matrix of Mahalanobis distances between species based on the two abiotic variables: mean annual temperature and annual precipitation. The level of correlation between matrices was assessed by means of Mantel tests with 9,999 random permutations as implemented in the *ade4* library (Dray & Dufour, 2007).

### 2.5 Environment‐morphology association

We performed phylogenetic generalized least squares (PGLS) to assess the relationship between climatic features (mean temperature and mean annual precipitation) and morphological traits while controlling for the influence of phylogeny. We also tested for differences in morphological traits among regions (four categories: Australia + Tasmania, Australia‐New Guinea, New Guinea and New Zealand + Chatham Islands), which greatly differ in mean temperature and annual precipitation values (both *p*‐values *p* < .001; Table S1), by means of phylogenetic ANOVA performed using the “phylANOVA” function (1,000 simulations) in *phytools* (Revell, 2012).

## 3 RESULTS

### 3.1 Among‐clade differences in morphological and climatic distances, and range overlap

Average morphological distances differed significantly among clades (subfamilies) but not in the expected direction (ANOVA; *F*_2,481_ = 48.67, *p *<* *.001). Morphological similarity among members of *Gerygone* was lower in comparison with that of members of Sericorninae, but higher with respect to the *Acanthiza* clade (Sericorninae: 2.01 ± 0.07; Gerygoninae: 1.41 ± 0.12; Acanthizinae: 1.38 ± 0.09). As expected, the extent of average range overlap within *Gerygone* was smaller in comparison with that of the two remaining clades (Sericorninae: 0.271 ± 0.39; Gerygoninae: 0.238 ± 0.34; Acanthizinae: 0.333 ± 0.40), but this relationship was not statistically significant (ANOVA; *F*_2,481_ = 1.86, *p *=* *.15). There were no significant differences in average climatic distances among groups (*p *>* *.75).

### 3.2 SURFACE analyses

We implemented a stepwise model‐fitting approach to estimate an adaptive landscape for functional morphology across the 53 species of acanthizid species examined. The final multi‐peak OU model included six regime shifts, two distinct regimes, and four convergent shifts (Figure 2). The AIC_c_ improved from 311.41 (AIC_c_ for the initial nonconvergent OU1 model) to 275.61 during the forward phase (ΔAIC_c_ = 35.8), then to a final AIC_c_ of 272.86 during the backward phase (ΔAIC_c_ = 2.75). The Brownian motion model was poorly supported compared with the SURFACE‐generated Hansen model (AICc = 323.08). As illustrated in Figure 2, all species except five were grouped into a single (ancestral) adaptive regime (peak I). Four of these five distinctive species converged into two separate peak shifts (peaks II and III). Peak II grouped the two acanthizid species with highest scores for PC1 (i.e., longer tarsus and longer tail) namely, *Gerygone igata*, endemic to New Zealand, and *Pycnoptilus floccosus*, a ground‐dwelling species endemic to southeastern Australia and part of the major clade that contains *Sericornis*. Peak III grouped the two acanthizid species with highest scores for PC2 (i.e., larger bill and longer tarsus) namely, *Origma solitaria*, a mainly terrestrial species strongly associated to exposed sandstone rock formations (and part of a clade that includes *Sericornis*), and New Guinean *Pachycare flavogriseum*, which is the basal acanthizid lineage. The aberrant *Gerygone albofrontata*, endemic to the Chatham Islands and thus, the most geographically restricted lineage, constitutes an independent selective regime (Peak IV). For the SURFACE model, the trait‐specific rate of adaptation (α) for PC1 and PC2 is 3.94 and 3.91 million per years, respectively. Converted to phylogenetic half‐life (*t*_1/2_), which translates into the time to move halfway from the ancestral state to an adaptive optimum, these correspond to about 0.17 Ma in both cases.

When performing SURFACE analyses within each of the three main clades, we obtained very similar results to those obtained across the complete phylogeny. Within the major clade comprising *Sericornis*,* P. flocossus* was identified again as distinctive regime whereas *O. solitaria* was placed together with the remaining scrubwren species (Figure 2a). When restricting our analysis to the genus *Acanthiza* clade, SURFACE identified a single‐species regime, not detected in the first (global) analysis, which included *A. murina* (Figure 2a). No regime shifts were evident within *Gerygone* (Figure 2a).

### 3.3 Ecomorphological diversification over time

The SURFACE model (OUM_SURFACE_) provided the best fit for the evolution of both PC axes in the Acanthizidae (Table 1). The multi‐peak model with six regimes defined by the geographic regions (OUM_region_) was the second most supported model in both cases (Table 1). Support for the Brownian motion (BM) and the early‐burst (EB) model was low (Table 1), suggesting that morphological evolution in this group is not driven by either a random‐walk or a niche‐filling process. Likewise, the AIC_c_ values obtained for the OU_niche_ model indicate that morphological disparity in acanthizids is not constrained by foraging mode (Table 1).

**Table 1:** Comparisons of six evolutionary model fit for the two principal components (PC1 and PC2) describing functional morphology in acanthizid species. A full description of each model is provided in the main text (see Section 2). ΔAICc is the model's mean AICc minus the minimum AICc between models

Model	Loglik	AIC_c_	ΔAIC_c_
PC1			
BM	−72.93	150.10	28.31
EB	−72.93	152.35	30.56
OU1	−71.52	149.53	27.74
OUM_niche_	−71.15	158.73	36.94
OUM_region_	−63.59	146.38	24.59
OUm_surface_	−54.00	121.79	0
PC2			
BM	−82.56	169.36	26.99
EB	−82.56	171.60	29.23
OU1	−75.64	157.77	15.40
OUM_niche_	−71.00	158.44	15.07
OUM_region_	−63.49	146.19	3.82
OUm_surface_	−64.29	142.37	0

### 3.4 Mantel tests

**Figure 3:**
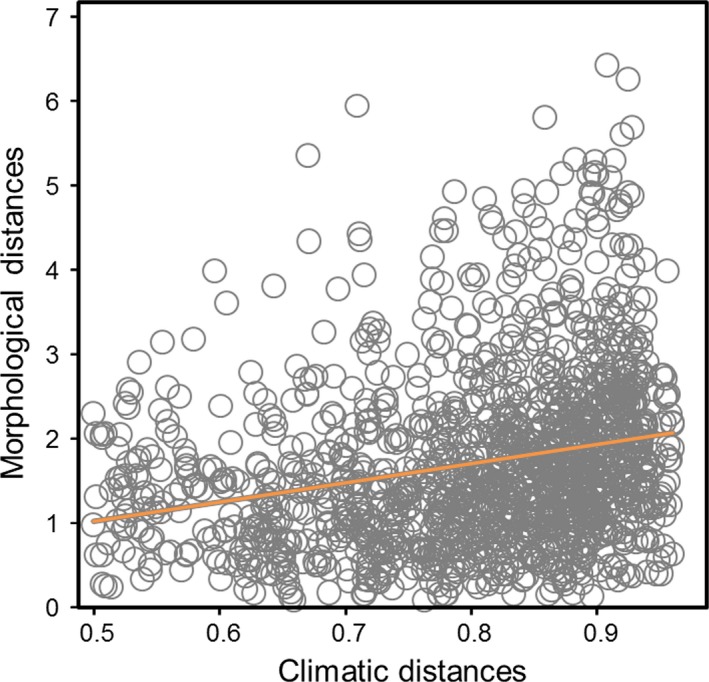
Morphometric distances among acanthizid species plotted against climatic distances

As expected due to common ancestry, morphologically similar species are more closely related than less similar species (*r *=* *.13, *p *=* *.026). However, we did not find a significant correlation between morphological distance and either range overlap (*r *=* *.03, *p *=* *.28) or geographic distance (*r* ~ 0, *p *=* *.72). That is, coexisting (sympatric) species do not exhibit greater phenotypic divergence than allopatric species. The extent of range overlap tended to increase with phylogenetic relatedness supporting the idea that spatially close species are more recently related that more spatially distant species, but the correlation was not statistically significant (*r *=* *.05, *p *=* *.074). Morphological and climatic distances were significantly correlated (*r *=* *.25, *p *=* *.005), indicating that species living in areas with the most distinct climatic conditions show more dissimilar morphotypes than species occupying areas with comparable climate (Figure 3). Geographic and phylogenetic distances were not significantly correlated (*r *=* *.03, *p *=* *.13).

### 3.5 Environment ‐morphology association

**Figure 4:**
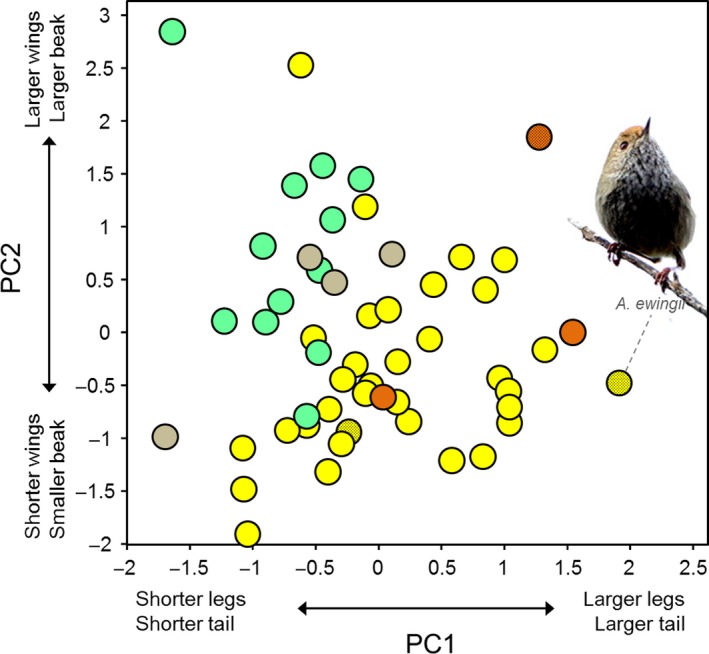
Scatterplot of acanthizids along the two principal component axes. Note: *Pycnoptilus floccosus* (Australia, PC1 = 4.334, PC2 = 0.591) was omitted from the graph for illustrative purposes. Dots are colored by geographic region according to the color coding shown in Figure 1

**Figure 5:**
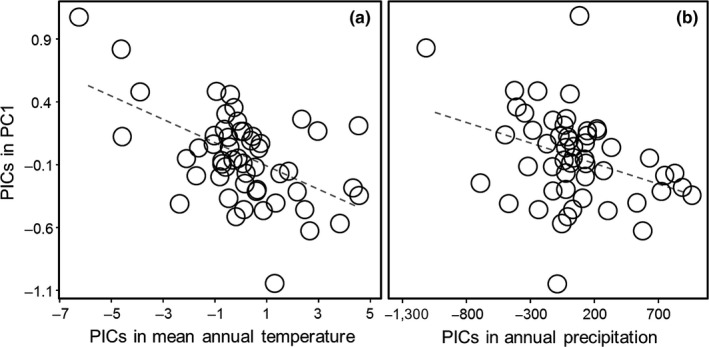
Relationship between the first principal component (PC1) and (a) mean annual temperature and (b) annual precipitation in acanthizid species represented in the form of standardized phylogenetic independent contrasts (PICs)

PGLS analyses revealed significant environment‐morphology associations within the acanthizids. We found significant negative correlations between species scores for PC1 and both temperature (PGLS; *r* ^2^ = .19, *F*_1,52_ = 14.09, *p *<* *.001; Figure 4a) and precipitation (PGLS; *r* ^2^ = .09, *F*_1,52_ = 6.56, *p *=* *.010; Figure 4b), whereas PC2 correlated positively with precipitation (PGLS; *r* ^2^ = .25, *F*_1,52_ = 18.81, *p *<* *.001). There was no significant relationship between PC2 and temperature after correcting for phylogeny (PGLS; *F*_1,52_ = 0.49, *p *=* *.38). Overall, these results indicate that species inhabiting more xeric environments have longer tarsi, longer tails, smaller beaks, and shorter wings. Accordingly, we observed that New Guinean species (i.e., species settled in wet and warm environments) tend to cluster together (regardless of the genus to which they belong or their foraging substrate) around the northwest sector of the morphospace, whereas most Australian species exhibit negative values for the PC2 axis (Figure 5). Those species whose distributional range comprises both regions show intermediate morphotypes between the New Guinean and the Australian types (Figure 5). The three highest values for PC2 are represented by insular endemics from Tasmania, New Zealand, and Chatham Islands (Figure 5). There were found marginally significant differences among regions for both morphological axes (PhylANOVA; PC1: *F *=* *5.34, *p *=* *.057; PC2: *F *=* *4.36, *p *=* *.071).

## 4 DISCUSSION

The Acanthizidae form a morphologically cohesive group and in contrast to our predictions, we did not find greater morphological variability in thornbills and scrubwrens (sympatric groups) in comparison with gerygones, a clade comprising fundamentally allopatric species. Our results indicate that species belonging to these three main groups overlap along the morphological space defined by the two axes, PC1 and PC2 (Figure S1). That is, no clade occupies a unique region of morphospace. It is striking as gerygones conform a suite of taxa specialized in obtaining their food by snatching it from the foliage, whereas thornbills and scrubwrens are more ecologically diverse (see Figure 2). Thus, despite almost all *Gerygone* species are canopy‐gleaning foragers, members of this clade have not developed a distinctive morphology in relation to this feeding strategy. This probably reflects the generalist nature of the acanthizid phenotype (see also Maestri, Patterson, Fornel, Monteiro, & de Freitas, 2016). Overall, gerygones exhibit a body form that is not substantially different from that of other members of the family. The high degree of morphological resemblance observed across the entire radiation is mirrored in the results yielded by the SURFACE algorithm. SURFACE did not detect convergence across lineages occupying equivalent foraging niches as has been shown in other groups such as dragon lizards, boas, terapontid fishes, and myobatrachid frogs (e.g., Collar, Schulte, O'Meara, & Losos, 2010; Davis & Betancur‐R, 2017; Esquerré & Keogh, 2016; Reynolds et al., 2016; Vidal‐García & Keogh, 2017). The only convergent regimes identified by SURFACE corresponded to monotypic genera and/or lineages that have colonized a novel and underexploited environment like *Origma solitaria*, the only acanthizid species strongly associated with rock formations. The SURFACE model (OU_SURFACE_) received substantial support in comparison with alternative models (BM, OU1), indicating that the identified cases of convergence were not incidental (Figure 2b). However, convergence is not necessarily the result of adaptation; it can arise as result of exaptation, correlated response to selection on another trait, or coincidence (Losos, 2011; Revell, Harmon, Langerhans, & Kolbe, 2007). On the other hand, *G. albofrontata*, endemic of the Chatham Islands and thus a highly restricted species, represent a distinctive regime that may have undergone divergent evolution recently following colonization by a small founder population from its closest relative *G. igata*, endemic to New Zealand (Ford, 1985). As this process has occurred relatively recently (about 1–1.5 Ma), we found that the optimum associated to the peak occupied by *G. albofrontata* fell outside the range of the trait data for PC1 and PC2 (Figure 2c; see also Ingram & Kai, 2014). Our results are thus congruent with Keast and Recher (1997) who examined the ecomorphology of gerygones and noted that the greatest evolutionary shifts (i.e., the greatest departures from its generalized features) occur in the insular species, *G. igata* and *G. albofrontata*. It suggests that islands, with their depauperate avifauna, allow insular species to expand into novel morphological space (i.e., underexploited adaptive zones) via ecological release, whereas the mainland forms may be subject to bounded phenotypic evolution as result of interspecific competition (Boucher & Démery, 2016). Thus, as character displacement is facilitated by “ecological opportunity,” it is possible that acanthizids have been prevented from occupying a wide region of the ecomorphospace by the presence of members of other insectivorous bird families (Pfennig & Pfennig, 2009).

Despite the expectation that the existence of direct competition between sympatric species promotes phenotypic divergence, we failed to find any correlation between morphometric distance and the degree of geographic overlap between acanthizid species. There are several alternative explanations for this result. Firstly, the set of morphological traits used in this study could not be capturing the relevant information needed to detect interspecific variation among acanthizid species. For instance, the phenotypic divergence could be along other axes of variation (such as bill depth or foot morphology) not considered here. Secondly, our overlap measurements may constitute a poor estimator of the real syntopy, being necessary field studies at community level (i.e., information on local assemblages, see e.g., Miller, Zanne, & Ricklefs, 2013; Miller et al., 2017) in order to address this question in detail. Thirdly, here we are assuming that range overlap is a surrogate of the potential for competition but direct interspecific interactions may be low in certain habitats as result of spatiotemporal segregation (e.g., altitude partitioning; Diamond, 1973). Lastly, resource availability could be high enough to allow coexistence of species without having to resort to a high degree of specialization as is the casein Darwin's finches (de León et al., 2014).

Results obtained using *OUwie* confirmed that acanthizid morphology is not shaped by foraging strategy. The multi‐peak model with regimes defined by foraging niche categories explained a smaller portion of the evolution of phenotypic variation than other multiple optima models. The second best‐fit model in both cases (for PC1 and PC2) was the model with distinctive adaptive regimes for each geographic region, which largely differ in terms of climate and predominant habitat types. For instance, the New Guinean region is dominated by rainforests which require high precipitation and warm temperatures, whereas Australia—with the exception of east coast and southwest forest and the coastal areas dominated by mangroves—is characterized by more xeric conditions and more open habitats. Accordingly, we observed that species belonging to each one of these regions (also those with a shared distribution, i.e., Australo‐Papuan lineages) are slightly differentiated across the space defined by our two morphological axes. New Guinean species tend to occupy the northwest sector of the morphospace, whereas most species inhabiting mainland Australia exhibit negative values for the PC2 axis (Figure 4). In line with this, we also found significant patterns when representing our PCs for morphology along climatic axes. We observed that species inhabiting wetter and warmer environments, such as rainforests, exhibit short tarsi, larger wings, and a longer bill. Relatively short legs may be advantageous to species that frequently perch on the canopy for gleaning, as is the case in most arboreal, small insectivorous birds (Miles & Ricklefs 1984; Kaboli, Aliabadian, Guillaumet, Roselaar, & Prodon, 2007; Leisler & Winkler, 1985; Osterhaus, 1962). Conversely, species inhabiting more arid environments, which are represented in the form of sclerophyllous woodlands and scrublands (mallee and mulga), tend to have longer tarsi, which is considered an adaptation for cursorial locomotion in open habitats, thereby increasing running speed and broadening the field of view (Grant, 1966; Leisler, Ley, & Winkler, 1989; Schon, 1998). Thus, there is evidence of some degree of morphological structuring in this taxonomic group at a broad scale. This finding is in agreement with the main assumption underlying ecogeographic rules; that is, morphological traits are related to climatic features (Millien et al., 2006). Such association does not necessarily need to be causal; morphological traits may vary directly (through physiological mechanisms) in response to climatic conditions and/or indirectly in relation to environmental factors (habitat structure, vegetation type etc.) which are shaped by climate (e.g., Gvoždík et al., 2008; Yom‐Tov & Geffen, 2006). Unfortunately, our correlative approach does not allow us to discern between these possibilities. Yet, what is clear from this study is that climate seems to be a more important predictor of morphology than is foraging niche or evolutionary relatedness of species in the Acanthizidae family (see also Luxbacher & Knouft, 2009). The hypothesis of climate‐driven morphological variation is supported by the existence of a statistically significant positive relationship between morphological and climatic distances (Figure 3). Such an association indicates that shared environmental conditions may lead to morphological resemblance even among nonclosely related species due to local selective pressures (Keast, 1978). In turn, it is likely that this effect had been buffered as consequence of the complex biogeographic history characteristic of this and other Australo‐Papuan bird radiations, whose evolution has taken the form of repeated interchanges between Australia and New Guinea (e.g., Christidis, Irestedt, Rowe, Boles, & Norman, 2011; Marki et al., 2017). Lack of congruence between acanthizid phylogeny and geographic distribution is illustrated in Figure 2, where it can be clear that New Guinean lineages are distributed along the three main clades. Accordingly, no correlation was found when regressing climatic distances onto phylogenetic distances (Mantel test; *r *=* *.03, *p *=* *.20) indicating that species disperse randomly through climate space. Thus, abiotic (temperature/precipitation) association of acanthizids shows no strong phylogenetic partitioning among major lineages as it would be expected if the colonization of new environments from the source area had occurred in one wave. If so, the level of phenotypic resemblance among members from the same region would probably have been greater (cf. Ingram & Kai, 2014).

Although it is traditionally assumed that competition in exploiting different trophic resources is a major force promoting morphological diversification among closely related groups (e.g., Schluter & McPhail, 1993), we failed to find a link between foraging habit and morphological attributes in acanthizids. Several factors may explain the lack of specialization in functional morphology between, for example, canopy‐gleaners and ground‐dwelling species. It could be argued that acanthizids have evolved an optimal (all‐purpose) morphology that allows them to perform relatively well (or similarly poorly) at several tasks; this has been previously described in some families of lizards with absence of ecomorphs (i.e., functionally intermediate or “jack of all trades and master of none” morphology; Arnold, 1998; see also Tulli et al., 2016; Maestri et al., 2017). Thus, conservation of a stereotyped morphology may represent an evolutionary mechanism by which species could exploit a variety of environments. In turn, shared environmental conditions might also promote morphological resemblance making difficult the appearance of morphological differentiation between closely related species. Thus, the fundamental niche seems to play a more important role than the realized niche in shaping morphological variation of acanthizids.

## CONFLICT OF INTEREST

None declared.

## AUTHOR CONTRIBUTIONS

VGN and LC conceived the study. PZM provided the phylogenetic tree. VGN and MRR performed all analyses. VGN wrote a first draft of the manuscript, and all coauthors significantly contributed to improve it up to the final version.
