# Supplementary material for: Environmental determinism, and not interspecific competition, drives morphological variability in Australasian warblers (Acanthizidae)
Source: Ecol Evol. 2018 Mar 23;8(8):3871–82. doi: 10.1002/ece3.3925 (PMC5916309; doi:10.1002/ece3.3925)

**Supporting Information**

Environmental determinism, and not interspecific competition, drive morphological variability in Australasian warblers (Acanthizidae)

Vicente García-Navas, Marta Rodríguez-Rey, Petter Z. Marki and Les Christidis

**Table S1**. Mean precipitation and mean annual precipitation values for each one of the four geographic regions inhabited by acanthizid species included in this study.

| Distributional range | Temperature (ºC) | Precipitation (mm year^-1^) |
| --- | --- | --- |
| Australia (including Tasmania) | 18.36 | 690.59 |
| New Guinea | 22.98 | 3071.85 |
| Australia and New Guinea | 24.55 | 2033.16 |
| New Zealand and Chatham Islands | 13.95 | 924.89 |

**Figure S1**. Morphospace projection of the two principal components (PC1 *vs*. PC2). Each dot represents one species (i.e. PC scores from the mean value for each species), colored according to (a) the subfamily to which they belong, and (b) their foraging strategy (see Fig. 1). Note: *P. floccosus* (Sericorninae, ground, PC1 = 4.334, PC2 = 0.591) was omitted from the graph for illustrative purposes.


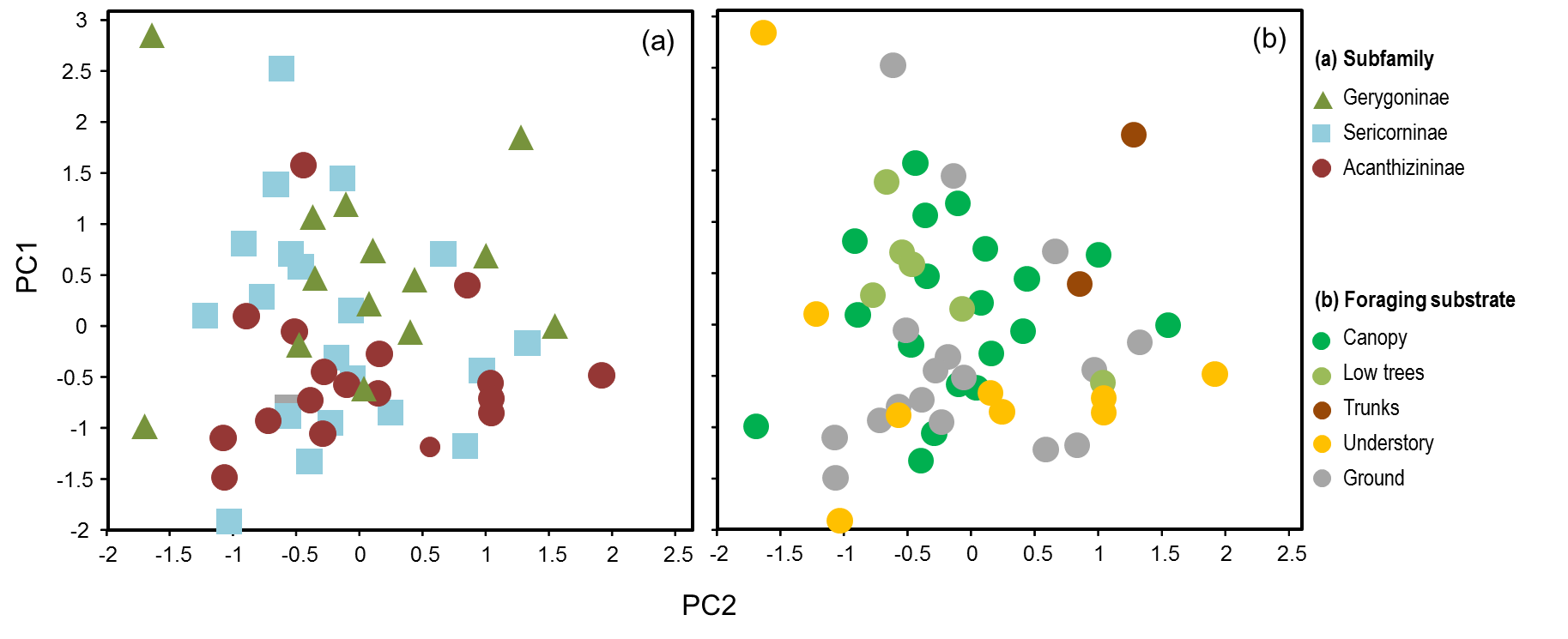

Supplement: Supplementary file 1 [file ECE3-8-3871-s001.docx]
